# Examining the relationship between birth weight and attention-deficit hyperactivity disorder diagnosis

**DOI:** 10.3389/fpsyt.2023.1074783

**Published:** 2023-05-24

**Authors:** Meng Ni, Lijuan Li, Wei Li, Qianqian Zhang, Jiuru Zhao, Qianwen Shen, Dongting Yao, Tao Wang, Baihe Li, Xiya Ding, Sudong Qi, Xiaoyi Huang, Zhiwei Liu

**Affiliations:** ^1^International Peace Maternity and Child Health Hospital, School of Medicine, Shanghai Jiao Tong University, Shanghai, China; ^2^International Peace Maternity and Child Health Hospital of China Welfare, Shanghai Jiao Tong University, Shanghai, China; ^3^Shanghai Key Laboratory of Embryo Original Disease, Shanghai, China

**Keywords:** ADHD, birth weight, adverse childhood experiences, family resilience, NSCH

## Abstract

**Background:**

Attention-deficit hyperactivity disorder (ADHD) is a neurodevelopmental condition that is prevalent in children worldwide. We evaluated the potential relationship between birth weight and ADHD using newly released data from the National Survey of Children’s Health 2019–2020.

**Methods:**

This population-based survey study used parent recollection data that were collected and submitted by 50 states and the District of Columbia to the National Survey of Children’s Health database from the National Survey of Children’s Health database. Those aged < 3 years and without birth weight or ADHD records were excluded. Children were stratified according to ADHD diagnosis and birth weight: very low birth weight (VLBW, < 1,500 g), low birth weight (LBW, 1,500–2,500 g), and normal birth weight (NBW, ≥ 2,500 g). Multivariable logistic regression was applied to examine the causal association between birth weight and ADHD while controlling for child and household characteristics.

**Results:**

The final sample consisted of 60,358 children, of whom 6,314 (9.0%) were reported to have an ADHD diagnosis. The prevalence of ADHD was 8.7% in NBW children, 11.5% in LBW, and 14.4% in VLBW. Compared with NBW children, LBW children [adjusted odds ratio (aOR), 1.32 (95% CI, 1.03–1.68)], and VLBW children [aOR, 1.51 (95% CI, 1.06–2.15)] had a significantly higher risk of ADHD after adjusting all variables. These associations persisted in the male subgroups.

**Conclusion and relevance:**

This study found that LBW and VLBW children were at a higher risk of ADHD.

## Introduction

Attention-deficit hyperactivity disorder (ADHD), characterized by a non-episodic pattern of inattentive, hyperactive, or impulsive symptoms, is one of the most prevalent developmental disorders among young children and adolescents (1). Approximately 2–7% of children and adolescents are estimated to be diagnosed with ADHD (2). ADHD has been extensively studied; however, its potential etiology is complicated. Heredity contributes to approximately 70% of ADHD (3). Significant genome-wide hits such as *FOXP2*, *SORCS3*, and *DUSP6* have been reported (4). Despite this, environmental factors are crucial and cause 10–40% of the variance related to ADHD (5). Numerous prenatal risk factors are reported to be associated with ADHD, including maternal emotional stress, substance exposure during pregnancy, prematurity, low birth weight (LBW), and several other complications during pregnancy, labor, and infancy (6). However, the cause-and-effect relationship between prenatal risk factors and the occurrence and prognosis of ADHD is unclear, as evaluated by previous reviews considering insufficient confounding factors (7).

Children with LBW seem to have more cognitive and psychiatric symptoms such as anxiety and depression (8). A meta-analysis found a higher ADHD risk in premature or LBW infants (9). However, LBW was not evaluated separately and heterogeneity could not be ignored. A cohort study from Japan including 796 children aged 8–9 years with completed follow-ups found that children with low birth weight (< 2,000 g) and genetic predisposition factors summarized from a large-scale genome-wide association study are prone to ADHD (10). However, several hypotheses have suggested that postnatal biological and social adversities contribute to increased vulnerability to ADHD (1). For example, ADHD and adverse childhood experiences (ACEs) co-occur at high frequency, as some ADHD symptoms may be a reaction to trauma (11). Children with ADHD were found to have a number of household characteristics that predispose them to moderate/severe ADHD; they also were probably to possess a higher proportion of ACEs compared to children without ADHD (12). On the contrary, resilience seems to be a protective factor, with which children tend to behave thriving in spite of challenges faced (13). Specifically, it could be drawn that resilience mitigated the negative impact of ACEs on children’s repeating grades and increased school engagement among children with chronic mental and physical health conditions by analyzing 2011–2012 NSCH data (14).

This study examined the relationship between LBW and very low birth weight (VLBW) and later outcomes of ADHD in 3–17-year-old children by analyzing cross-sectional data from the National Survey of Children’s Health (NSCH) survey cycles 2019 and 2020, which are nationally representative and population-based. Then, we performed subgroup analysis to explore the potential effect of children’s characteristic and postnatal and social factors like adverse childhood events and family resilience on the association between birth weight and ADHD.

## Materials and method

### Study population

**FIGURE 1:**
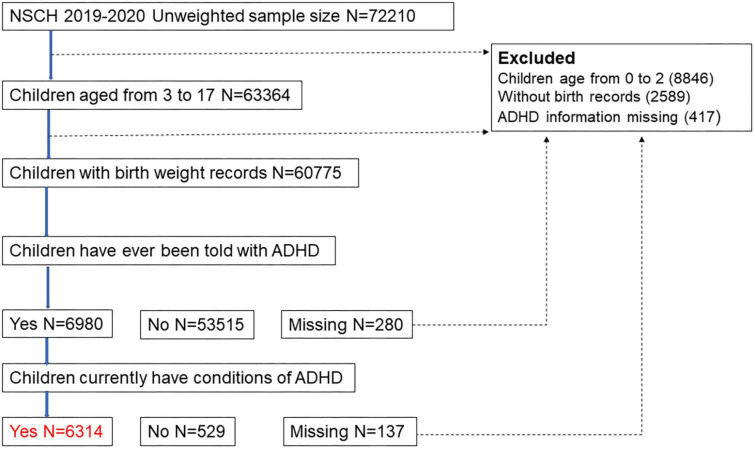
The flow chart of the experiment.

Combined 2019–2020 data from the NSCH database were used in this cross-sectional survey (15). The NSCH survey was conducted via mail and online by the Data Resource Center (DRC) to collect health information on children and adolescents. Survey respondents were parents or caregivers with at least one child aged 0–17 years who resided in the same household during the entire interview. A total of 72,210 surveys were completed nationwide from June 2019 to January 2021. Children under 3 years of age were excluded. Surveys without information on ADHD or birth weight were excluded. Ultimately, 60,358 children aged 3–17 years were included in this study (Figure 1).

Birth weight was assessed as a continuous variable. However, in the released data, the birth weight of the population was truncated by 72 and 155 ounces (2,041–4,394 g), respectively, where children born under 2,041 g were considered 2,041 g and over 4,394 as 4,394 g. Therefore, to avoid uncertainty, an association between ADHD and birth weight was estimated using birth weight data between 73 and 154 ounces (2,070–4,366 g).

The International Peace Maternity and Child Hospital Institutional Review Board deemed the study exempt from review and waived the informed consent requirement because the data used were publicly available and the study did not involve human participants. We followed the strengthening the reporting of observational studies in epidemiology (STROBE) reporting guidelines.

### Outcome and exposure

The primary outcome of interest was the diagnosis of ADHD in children. The ADHD diagnosis was identified using the following two NSCH questions:

1. “Has a doctor or other healthcare provider ever told you that this child has Attention-Deficit Disorder or attention-deficit hyperactivity disorder, that is, ADD or ADHD?”
2. “Does this child currently have the condition?”

Attention-deficit hyperactivity disorder (ADHD) was confirmed if the parents or caregivers responded “yes” to the two questions above, where the children were deemed as currently having ADHD.

### Covariates

The covariates in the model included child and household characteristics. Child characteristics included sex, age, and race/ethnicity. According to the survey questions, the children were grouped into preschool children (3–5 years of age), school-aged children (6–12 years of age), and adolescent children (13–17 years of age). Race/ethnicity was classified as white, Hispanic, African-American, Asian, or other.

Caregivers’ characteristics included poverty/income level and health insurance status. Poverty/income levels were 0–99%, 100–199%, 200–399%, and 400% or above federal poverty level. The NSCH prepares nine questions on ACEs, including parental separation/divorce, parental death, witnessing household violence, witnessing neighborhood violence, household mental illness, household incarceration, household substance abuse, racial/ethnic mistreatment, and economic hardships. In this study, ACEs were categorized into three groups: no ACE, one ACE, and two or more ACEs. Family resilience includes family members: (1) discussing what to do, (2) working together to solve the problem, (3) knowing they have whom they can draw strength from, and (4) staying hopeful even in difficult times when the family faces problems. Families with 0–1 item, 2–3 items, and all four items were labeled as some/none of the time, most of the time, and all of the time, respectively. The household smoking status was determined by “Does anyone living in this child’s household use cigarettes, cigars, or pipe tobacco?” as “Yes” or “No.” When necessary, a missing category for the covariates was added. According to the official guidelines for data analysis,^1^ the proportion of missing values is less than 2% in most cases. The elimination of these values did not change the estimated prevalence and only slightly affected the weighted population counts.

### Statistical analysis

The 2019–2020 combined dataset consisted of two individual survey rounds (2019 and 2020 NSCH). Only items with the same unique household identifier (HHID) across both datasets are included in the DRC-produced combined dataset. Information on the process of the datasets combination and weight adjustments is available in the “Guide to Analysis of Multi-Year NSCH Data” (15). Each record in the individual 2019 and 2020 NSCH public release datasets was assigned a single sampling weight for data analysis accuracy purposes. In the combined 2019–2020 dataset, each survey weight has been divided by two (FWC/2) from the original sampling weight (FWC), which is not included in the combined dataset, named “fwc_1920” in the combined dataset.

The weighting process was designed to estimate national- and state-level prevalence for a variety of child and family health measures with an effective and representative sample size (15). In the weighting process, each household interviewed in the sample was assigned a baseline weight and adjusted for the non-response rate after selection screening. The eligible children from the screener interview cases were then adjusted to population controls, and labeled with HHID. Then, the sampling weight was adjusted to topical non-response and each state’s weighted survey responses to selected characteristics of the state’s population of non-institutionalized children aged 0–17. Characteristics included household size, household poverty threshold, educational attainment of the household respondent, race, ethnicity, and special health care needs status by state, as well as national age and sex distributions. The survey sampling plan is set up in R with the separate “survey” package as follows: strata: FIPSST (state of residence) and STRATUM (identifies households with children); cluster: HHID (unique household identifier); weight: fwc_1920.

After the sample weighting process, estimates of the population characteristics were reported with 95% CI, based on Wald’s method. Categorical variables were reported as frequencies and percentages. Additional information is available on the DRC website (see text footnote 1). All *P*-values were 2-sided, and *P* < 0.05 was statistically significant.

Based on logistic regression models, we estimated the odds ratios (ORs) with 95% CIs of birth weight vs. ADHD. The main analyses examined the association between birth weight (LBW and VLBW groups separately) and ADHD, using normal birth weight (NBW) children as the reference group. In light of previous research, we considered multiple confounding factors using three models. Model 1 was unadjusted, while Model 2 was adjusted for sociodemographic characteristics such as age, race, ethnicity, poverty/income level, and health insurance status. Additionally, Model 3 was adjusted for the variables in Model 2 plus preterm birth to estimate the independent effect of birth weight on ADHD. Restricted cubic spline with 3 knots was applied to flexibly model and visualize the relation of predicted birth weight and ORs of ADHD. Furthermore, we performed the exploratory subgroup analysis where the population was stratified by preterm birth, age, sex, and race of the child, household smoking exposure, ACEs and family resilience based on interaction analysis and previous research. The widths of the CI was not adjusted for multiple testing, therefore, the results might not be used to statistical inference. Specifically, children were divided by ACEs status and family resilience to control the influence of the postnatal environment. All analyses were performed using the “survey” package in R 4.0.6.

## Results

The exposure and outcome variables for 60,358 children aged 3–17 years from the combined 2019–2020 NSCH sample, which included 72,210 children, were obtained. In our sample, 6,314 or 9.0% (95% CI 8.5, 9.4) were diagnosed with ADHD currently. Moreover, 796 or 1.3% (95% CI 1.2, 1.5) were reported to have VLBW, and 4,373 or 7.9% (95% CI 7.4, 8.4) were reported to have LBW (Table 1). The largest percentage of our sample consisted of children aged 6–12 years old (46.5%), while 19.7% were 3–5 years, and 33.8% were 13–17 years. Approximately half of our sample was male (51.2%) and white (50.7%). A total of 11.5% were preterm births, 17.4% of household poverty levels were under 100%, 6.9% were without insurance, and 14.3% had been exposed to secondhand smoke. Of the children, 20.2% had two or more ACEs, and 16.2% of families showed no resilience. Children with ADHD were also reported higher incidences of LBW (10.1 vs. 7.7%), VLBW (2.2 vs. 1.3%), and preterm birth (14.7 vs. 11.2%). Besides, increased age of children, male (71.1 vs. 49.2%), white or black race, lower household poverty level, household smoking exposure (21.3 vs. 13.6%), and poor maternal or paternal mental health had a higher incidence of ADHD. Furthermore, children with ADHD face more ACEs and poorer family resilience (Table 1).

**TABLE 1:** Population characteristics by ADHD diagnosis.

	Total		ADHD	No ADHA			
**Characteristic**	***N*%^a^**	**95 CI%**	***N*%^a^**	**95 CI%**	***N*%^a^**	**95 CI%**	***P* ^b^**
Population	60,358 (100)	–	6,314 (9.0)	8.5–9.4	54,044 (91.0)	90.6–91.5	
Birth weight (g)							<0.001
<1500	796 (1.3)	1.2–1.5	123 (2.2)	1.6–2.8	673 (1.3)	1.1–1.5	
1500–2500	4,373 (7.9)	7.4–8.4	546 (10.1)	8.6–11.8	3,827 (7.7)	7.2–8.2	
>2500	55,189 (90.8)	90.2-91.3	5,595 (87.7)	86.0–89.3	49,544 (91.1)	90.5–91.6	
Preterm birth							<0.001
Yes	6,512 (11.5)	10.9–12.1	959 (14.7)	13.2–16.3	5,553 (11.2)	10.5–11.8	
No	53,097 (88.5)	87.9–89.1	5,278 (85.3)	83.7–86.8	47,819 (88.8)	88.2–89.5	
Missing	749	–	77	–	672	–	
Age of child (years)							<0.001
3–5	11,023 (19.7)	19.0–20.5	193 (5.2)	4.0–7.0	10,830 (21.2)	20.4–22.0	
6–12	25,074 (46.5)	45.6–47.4	2,914 (50.6)	48.0–53.1	22,160 (46.1)	45.1–47.1	
13–17	24,261 (33.8)	32.9–34.6	3,207 (44.2)	41.7–46.7	21,054 (32.7)	31.9–33.6	
Sex of child							<0.001
Male	31,192 (51.2)	50.3–52.1	4,332 (71.1)	68.9–73.2	26,860 (49.2)	48.3–50.2	
Female	29,166 (48.8)	47.9–49.7	1,982 (28.9)	26.8–31.1	27,184 (50.8)	49.8–51.7	
Race of the child							<0.001
Hispanic	7,605 (25.5)	24.5–26.5	661 (19.8)	17.3–22.6	6,944 (26.0)	25.0–27.1	
White	41,178 (50.7)	49.8–51.5	4,652 (57.5)	54.8–60.1	41,930 (50.0)	49.1–50.9	
Black/African American	3,954 (13.3)	12.6–13.9	436 (15.5)	13.5–17.7	3,841 (13.0)	12.4–13.7	
Asia	3,019 (4.4)	4.1–4.7	90 (1.3)	0.9–2.1	2,929 (4.7)	4.4–5.1	
Other	4,602 (6.2)	5.8–6.6	475 (5.9)	5.0–6.9	4,127 (6.2)	5.8–6.6	
Household poverty level (% FPL)							<0.001
<100%	6,946 (17.4)	16.6–18.2	969 (21.8)	19.6–24.2	5,977 (17.0)	16.2–17.8	
100–199%	9,984 (21.5)	20.6–22.3	1,160 (22.9)	20.8–25.3	8,824 (21.3)	20.4–22.2	
200–399%	18,719 (29.8)	28.9–30.6	1,900 (27.8)	25.6–30.1	16,819 (30.0)	29.1–30.8	
=400%	24,709 (31.4)	30.6–32.1	2,285 (27.4)	25.5–29.5	22,424 (31.8)	31.0–32.6	
Insurance status							<0.001
Yes	57,313 (93.1)	92.6–93.6	6,041 (95.3)	94.1–96.3	51,272 (92.9)	92.3–93.5	
No	2,874 (6.9)	6.4–7.4	252 (4.7)	3.7–5.9	2,622 (7.1)	6.5–7.7	
Missing	171	–	21	–	150	–	
Household smoking exposure							<0.001
Yes	8,255 (14.3)	13.7–14.9	1,254 (21.3)	19.3–23.5	7,001 (13.6)	13.0–14.3	
No	50,936 (85.7)	85.1–86.3	4,943 (78.7)	76.5–80.7	45,933 (86.4)	85.7–87.0	
Missing	1,167	–	117	–	1,110	–	
Maternal mental health							<0.001
Excellent	38,780 (72.6)	71.7–73.4	3,191 (59.5)	56.8–62.1	35,589 (73.8)	72.9–74.7	
Good	11,640 (21.4)	20.6–22.2	1,597 (28.8)	26.4–31.4	10,043 (20.7)	19.9–21.6	
Fair	3,148 (6.0)	5.6–6.5	635 (11.7)	10.1–13.5	2,513 (5.5)	5.0–6.0	
Missing	6,790	–	891	–	5,899	–	
Paternal mental health							<0.001
Excellent	36,572 (78.7)	77.9–79.5	2,959 (69.5)	66.8–72.1	33,613 (79.5)	78.6–80.3	
Good	8,482 (17.1)	16.4–17.9	1,054 (23.6)	21.3–26.1	7,428 (16.6)	15.9–17.4	
Fair	1,918 (4.1)	3.7–4.6	319 (6.9)	5.7–8.3	1,599 (3.9)	3.5–4.4	
Missing	13,386	–	1,982	–	11,404	–	
ACEs							<0.001
No	35,478 (57.2)	56.3–58.1	2,455 (36.7)	34.3–39.2	33,023 (59.2)	58.2–60.2	
One	12,730 (22.7)	21.9–23.4	1,508 (25.5)	23.4–27.8	11,222 (22.4)	21.6–23.2	
Two or more	11,237 (20.2)	19.4–20.9	2,258 (37.7)	35.3–40.3	8,979 (18.4)	17.6–19.2	
Missing	913	–	93	–	820	–	
Family resilience							<0.001
Yes	49,816 (83.8)	83.1–84.5	4,747 (75.7)	73.4–77.9	45,069 (84.6)	83.9–85.3	
No	9,106 (16.2)	15.5–16.9	1,438 (24.3)	22.1–26.6	7,668 (15.4)	14.7–16.1	
Missing	1,436	–	129	–	1,307	–	

^a^Actual cases and weighted percentage.

^b^Rao Scott adjusted Pearson chi-square.

However, current ADHD diagnosis (VLBW, 14.4%; LBW, 11.5%; NBW, 8.7%), preterm birth (VLBW, 82.8%; LBW, 59.0%; NBW, 6.3%), sex (male, VLBW: 45.3%, LBW: 47.9%, NBW: 51.6%), race, household poverty level, exposure to secondhand smoke, and ACEs (two or more, VLBW: 26.0%, LBW: 24.6%, NBW: 19.7%) were associated with the birth weight of children (*P* < 0.05) (Table 2).

**TABLE 2:** Population characteristics by birth weight.

	< 1,500 (g)		1,500–2,500 (g)		≥ 2,500 (g)		
**Characteristic**	***N*%^a^**	**95 CI%**	***N*%^a^**	**95 CI%**	***N*%^a^**	**95 CI%**	***P* ^b^**
Population	796 (1.3)	1.2–1.5	4,373 (7.9)	7.4–8.4	55,189 (90.8)	90.2–91.3	
ADHD							< 0.001
Yes	123 (14.4)	10.8–18.8	546 (11.5)	9.8–13.4	5,645 (8.7)	8.2–9.1	
No	673 (85.6)	81.2–89.2	3,827 (88.5)	86.6–90.2	49,544 (91.3)	90.9–91.8	
Preterm birth							< 0.001
Yes	676 (82.8)	75.4–88.3	2,567 (59.0)	55.9–62.1	3,269 (6.3)	5.8–6.8	
No	108 (17.2)	11.7–24.6	1,745 (41.0)	37.9–44.1	51,246 (93.7)	93.2–94.2	
Missing	6	–	61	–	674	–	
Age of child (years)							0.493
3–5	133 (15.6)	11.4–21.0	771 (20.7)	18.2–23.5	10,119 (19.7)	18.9–20.5	
6–12	357 (51.2)	44.3–58.0	1,847 (45.1)	41.9–48.4	22,870 (46.6)	45.6–47.5	
13–17	306 (33.2)	27.5–39.4	1,755 (34.1)	31.1–37.1	22,200 (33.7)	32.9–34.6	
Sex of child							0.022
Male	379 (45.3)	38.7–52.2	2,054 (47.9)	44.7–51.1	28,759 (51.6)	50.6–52.5	
Female	417 (54.7)	47.9–61.3	2,319 (52.1)	48.9–55.3	26,430 (48.4)	47.5–49.4	
Race of the child							< 0.001
Hispanic	107 (24.6)	17.7–33.1	625 (23.9)	20.6–27.5	6,873 (25.6)	24.6–26.7	
White	452 (42.4)	36.1–48.9	2,559 (41.4)	38.5–44.4	38,167 (51.6)	50.6–52.5	
Black/African American	119 (22.4)	17.5–28.2	485 (20.6)	18.1–23.3	3,350 (12.5)	11.8–13.2	
Asia	53 (4.9)	2.7–9.0	331 (6.5)	5.1–8.2	2,635 (4.2)	3.9–4.6	
Other	65 (5.7)	3.9–8.4	373 (7.6)	6.0–9.5	4,163 (6.1)	5.7–6.4	
Household poverty level (% FPL)							0.003
< 100%	146 (27.1)	20.3–35.2	630 (20.0)	17.4–22.8	6,170 (17.0)	16.2–17.9	
100–199%	154 (19.2)	14.8–24.4	806 (23.0)	20.0–25.5	9,024 (21.4)	20.5–22.3	
200–399%	237 (26.9)	21.8–32.7	1,324 (28.6)	25.9–31.8	17,158 (29.9)	29.0–30.8	
≥ 400%	259 (26.7)	21.6–32.9	1,613 (28.6)	26.0–31.4	22,837 (31.7)	30.9–32.5	
Insurance							0.732
Yes	744 (91.1)	83.8–95.3	4,131 (93.0)	91.0–94.5	52,438 (93.2)	92.6–93.7	
No	47 (8.9)	4.7–16.2	226 (7.0)	5.5–9.9	2,601 (6.8)	6.3–7.4	
Missing	5	–	16	–	150	–	
Household smoking exposure							0.007
Yes	132 (20.0)	14.3–27.4	729 (16.9)	14.7–19.4	7,457 (14.0)	13.4–14.7	
No	656 (80.0)	72.6–85.7	3,596 (83.1)	80.6–85.3	47,012 (86.0)	85.3–86.6	
Missing	8	–	48	–	720	–	
ACEs							0.002
No	415 (50.8)	43.7–57.8	2,344 (53.7)	50.5–56.9	32,719 (57.6)	56.6–58.5	
One	188 (23.2)	18.2–29.2	971 (21.7)	19.4–24.2	11,571 (22.7)	21.9–23.6	
Two or more	179 (26.0)	19.6–33.5	980 (24.6)	21.8–27.7	10,078 (19.7)	18.9–20.5	
Missing	14	–	78	–	821	–	
Family resilience							0.234
Yes	640 (84.1)	79.1–88.2	3,534 (82.1)	79.4–84.4	45,642 (83.9)	83.2–84.6	
No	132 (15.9)	11.8–20.9	718 (17.9)	15.6–15.4	8,256 (16.1)	15.4–16.8	
Missing	24	–	121	–	1,281	–	

^a^Actual cases and weighted percentage were presented.

^b^Rao Scott adjusted Pearson chi-square.

Thereafter, we assessed the association between birth weight and ADHD and discovered that VLBW was associated with a higher risk of the disorder [unadjusted odds ratio (uOR) 1.77, 95% CI 1.28–2.45], and a similar trend was noted for LBW (uOR 1.37, 95% CI 1.13–1.65) (Table 3). After adjusting for age, race, sex, household poverty level, exposure to secondhand smoke, and insurance status (Model 1), the odds of ADHD were 83% higher in the VLBW [adjusted odds ratio (aOR), 1.83 (95% CI, 1.32–2.55)] group and 43% in LBW group [aOR, 1.43 (95% CI, 1.17–1.74)] than in the normal weight group. Since preterm birth was accompanied by lower birth weight in most cases, we adjusted for preterm birth in Model 2, and the association remained [VLBW: aOR, 1.58 (95% CI, 1.11–2.27); LBW: aOR, 1.32 (95% CI, 1.04–1.67)]. The following risk factors were also associated with ADHD significantly: male sex, older age, white race, lower household poverty level, more ACEs, and no family resilience; the details of the model are listed in Supplementary Table 1.

**TABLE 3:** Odds ratios for the associations between birth weight and ADHD.

	Unadjusted model		Model 1^a^		Model 2^b^	
**Birth weight**	**OR**	**95% CI**	**OR**	**95% CI**	**OR**	**95% CI**
NBW	Reference		Reference		Reference	
LBW	1.37	1.13–1.65	**1.43**	1.17–1.74	**1.32**	1.04–1.67
VLBW	**1.77**	1.28–2.45	**1.83**	1.32–2.55	**1.58**	1.11–2.27

^a^Model 1 was adjusted for age, sex, and race, family of the children, household poverty, health level, insurance, status, household smoking, parental mental health exposure.

^b^Model 2 was adjusted for preterm birth and the variables in Model 1.

The bold value indicated that the *P* value of the odds ratio was <0.05.

NBW, normal birth weight; LBW, low birth weight; VLBW, very low birth weight.

After adjustment for the same covariables in Model 2, a one-ounce (28 g) increase in birth weight was associated with a one percent reduction in the odds of ADHD [aOR, 0.99 (95% CI, 0.99–1.00), *P* = 0.005] (Supplementary Table 2). In addition, we used a restricted cubic splines model with three knots to estimate the non-linear relationship between birth weight and the OR of ADHD (Supplementary Figure 1). In this study, the optimal point of birth weight was 122.25 ounces (3,466 g) in the unadjusted model, whether too low or too high would result in an increased risk of ADHD. However, after adjusting for variables, the curve was a monotone decrease, and lower birth weight was associated with a higher risk of ADHD. Moreover, increased birth weight had less impact on ADHD compared with lower birth weight [2,750 g: aOR, 1.16 (95% CI, 1.09–1.23); 4,250 g: aOR, 0.97 (95% CI, 0.88–1.07); 3,500 g: reference]. the estimation of the model was listed in Supplementary Table 3.

Explorative stratified analyses by preterm birth, age, race, exposure to secondhand smoke, insurance status, ACEs, and family resilience were performed (Table 4).

**TABLE 4:** Subgroup analysis for the association of birth weight with ADHD.

	Birth weight			
	**NBW**	**LBW**	**VLBW**	
		**OR (95% CI)^a^**	**OR (95% CI)^a^**	***P* (interaction)**
Preterm birth	Reference			0.064
Yes		1.20 (0.90–1.59)	**1.64** (1.11–2.44)	
No		**1.44 (1.00–2.07)**	0.44 (0.14–1.40)	
Age of child (years)	Reference			0.024
3–5		2.80 (0.87–9.03)	0.26 (0.04–1.60)	
6–12		1.25 (0.92–1.7)	1.53 (0.94–2.50)	
13–17		1.21 (0.85–1.72)	**1.79** (1.05–3.06)	
Sex of child	Reference			0.473
Male		**1.44 (1.09–1.91)**	**1.67** (1.07–2.60)	
Female		1.11 (0.69–1.76)	1.42 (0.77–2.62)	
Race of the child	Reference			0.257
Hispanic		1.93 (0.95–3.92)	2.11 (0.71–6.32)	
White		1.05 (0.82–1.33)	1.13 (0.72–1.79)	
Black/African American		1.59 (0.97–2.61)	**2.28** (1.01–5.15)	
Asia		1.33 (0.28–4.53)	0.35 (0.02–5.36)	
Other		0.59 (0.29–1.17)	1.47 (0.47–4.61)	
Household smoking exposure	Reference			0.012
Yes		0.79 (0.52–1.20)	1.71 (0.75–3.90)	
No		**1.44** (1.10–1.89)	**1.63** (1.09–2.42)	
ACEs^b^	Reference			< 0.001
No		1.38 (0.87–2.21)	1.51 (0.80–2.87)	
One		1.37 (0.92–2.05)	**2.02** (1.04–3.92)	
Two or more		1.12 (0.80–1.58)	1.31 (0.71–2.41)	
Family resilience^b^	Reference			< 0.001
Yes		1.27 (0.97–1.67)	1.26 (0.85–1.88)	
No		1.31 (0.84–2.05)	**2.55** (1.13–5.73)	

^a^The model was adjusted for preterm birth, age, sex, race, household poverty level, health insurance, and household smoking exposure.

^b^Subgroup analysis based on ACEs or family resilience levels to provide an estimated unadjusted and adjusted prevalence odds ratios (uPOR and aPOR, respectively) and Wald 95 % CI.

The bold value indicated that the P value of the odds ratio was <0.05.

NBW, normal birth weight; LBW, low birth weight; VLBW, very low birth weight.

## Discussion

In this nationwide population-based survey study, we found that birth weight and both LBW and VLBW were significantly associated with ADHD compared to NBW after adjusting for preterm birth and other covariates.

Previous studies have indicated that birth weight may correlate with ADHD. Aarnoudse-Moens et al. (16) conducted a meta-analysis to examine academic achievement, behavioral problems, and executive function, and found that attention problems measured by teachers and parents using the child behavior checklist (CBCL) or teacher report form were more prevalent in very preterm (≤ 33 weeks’ gestation) and/or VLBW (≤ 1,500 g) children than in term and/or NBW children. Moreover, the laggard neurodevelopment of these children persists in young adulthood. Besides, a gradient correlation has also been found between prematurity or low birth weight and ADHD (17, 18). Considering that prematurity and low birth weight occur simultaneously in most cases, most studies have focused on the effect of LBW on ADHD in combination with prematurity or prematurity alone, as most studies did. According to recent studies, prematurity increases the risk of ADHD, and the risk increases with decreasing gestational age. Evidence from a population-based case-control study in West Australia (*N* = 12,991) found that mothers of children with ADHD were more likely to be delivered early, irrespective of the sex of the child, while low birth weight, post-term pregnancy, small for gestational age infants, fetal distress, and low Apgar scores were not identified as risk factors (19). However, the association between gestational age and ADHD has not been replicated in a US study (20). Similarly, children with ADHD (*N* = 2,243) were not more likely to be born prematurely than controls (*N* = 5,631) in a US case-control study (21). To eliminate the influence of preterm birth, a population-based study of term birth weight and its link to a range of neurodevelopmental outcomes using Norwegian health registries restricted our analyses to 1.8 million singleton babies born during a narrow range of gestational ages (39–41 weeks), which is a powerful predictor of a wide variety of neurodevelopmental disabilities independent of preterm delivery. Using the category of 3.5–3.9 kg as the reference, the odds reached for ADHD (2.1–5.4) at the smallest weights (22). Furthermore, Halmøy et al. (19) used data from Norwegian national registries, which comprised all adults born between 1967 and 1987 (*n* = ∼1.17 million), and found that among individuals born at term (week 37–41 of gestation), the risk of ADHD increased with decreasing Z scores of birth weight by gestational age. Therefore, there is a distinctive and pervasive pattern of neurodevelopmental morbidity associated with birth weight at term. In our research, after adjusting for maternal age, maternal education, marital status, year of childbirth, sex, and all pregnancy/labor/newborn factors, especially preterm birth, we indicated that lower birth weight may independently increase the risk for ADHD [VLBW: aOR, 1.58 (95% CI, 1.11–2.27); LBW: aOR, 1.32 (95% CI, 1.04–1.67)]. Furthermore, the association remained in LBW [aOR, 1.44 (95% CI, 1.00–2.07)] but not in VLBW. The potential reason might be that VLBW limited sample size [*n* = 108, 17.2%, (95% CI, 11.7–24.6%)]. In our study, preterm was not associated with ADHD after adjusting for all the variables [(Model 2: aOR), 1.16 (95% CI, 0.97–1.39)]. However, this might be due to the lack of analysis on the cause and extent of preterm births because of limited information (the value of gestational age is not available in the NSCH database). Further research is needed to address this issue.

Several hypotheses have indicated that environmental adversities during the fetal and postnatal periods, as well as parental genetic factors and pathological issues, such as hypothalamic-pituitary-adrenal (HPA) axis dysregulation and perinatal systemic inflammation, are causally related to neural disorders such as ADHD and other psychiatric and developmental disorders (23–25). Previous studies have demonstrated that traumatic household experiences have a crucial impact on the occurrence and later outcomes of ADHD (12). Furthermore, family resilience may play the role of a protective factor associated with reducing conduct disorders despite experiencing adversities in childhood (26). In our study, children with ADHD faced more ACEs and poorer family resilience components, indicating a potential correlation between ADHD and environmental factors. Nonetheless, longitudinal studies of children, including ACEs’ family resilience and ADHD, are needed to elucidate the causality. Other prenatal risk factors and the etiology of ADHD include maternal substance use in pregnancy, such as alcohol (OR = 2.8–11.7) (27, 28), tobacco (OR = 2.4) (29, 30), and environmental toxins exposure (31). In the current study, maternal smoking was a risk factor for ADHD [aOR, 1.52 (95% CI, 1.31–1.76)]. In the subgroup analyses, however, the adverse effects of LBW [aOR, 1.44 (95% CI, 1.10–1.61)] and VLBW [aOR, 1.63 (95% CI, 1.09–2.42)] were only observed in the group without household exposure. This indicates the complicated biology of tobacco in pre- and postnatal environments.

Finally, this study also provided the current national prevalence of ADHD, where 9.0% of US children had ADHD, which is close to prior estimates of the national prevalence of ADHD of 9.6% in 2017–2018 and 9.4% in 2016 from the NSCH data (12, 32).

### Strengths and limitations

This study utilized a nationally open-access representative sample to analyze and provide generalizable results for preschool-aged children in the US. The NSCH has been updated since 2015, using several methods, such as an address-based sampling frame, to decrease sample bias and include accommodation to improve the response rate. With a large NSCH dataset, several variables were adjusted for confounding factors, thus avoiding the limitations of many previous studies and providing a relatively solid result with large sample size.

However, this study has some limitations. First, ADHD diagnosis and severity information were collected from caregivers’ reports, which could not be confirmed with a healthcare provider and were inevitably subject to potential caregiver bias. And the bias might lead to the inconsistent results in stratified analysis of children with and without ACE. In addition, both inherited and environmental factors contribute to ADHD (33, 34); however, molecular genetic effects were not evaluated in this study. Third, research suggests an important overlap between ADHD and other neurodevelopmental problems, such as autism spectrum disorders (ASD) in genetics and symptoms (35), and children with ASD were not excluded from our study.

## Conclusion

In summary, our study results indicate that LBW/VLBW children, compared to the NBW group, were associated with subsequent ADHD diagnosis in 3–17-year-old children in the US. This study provides the latest nationally representative estimates of multiple ADHD diagnoses across the pediatric population in the US. Further investigations are necessary to elucidate the mechanisms underlying the association between LBW/VLBW infants and the risk of ADHD, as well as the interactions of birth weight with postnatal and environmental factors such as ACEs and family resilience to explore how other independent variables, particularly those having a biological basis, may work together with birth weight to impact ADHD in children.

## Data availability statement

Publicly available datasets were analyzed in this study. This data can be found here: http://www.childhealthdata.org/NSCH.

## Ethics statement

The studies involving human participants were reviewed and approved by the International Peace Maternity and Child Hospital Institutional Review Board. They deemed the study exempt from review and waived the informed consent requirement because the data used were publicly available, and the study did not involve human participants. Written informed consent to participate in this study was provided by the participants’ legal guardian/next of kin.

## Author contributions

MN, LL, and ZL: study design. MN, QS, WL, XD, SQ, TW, BL, and DY: data analyses and interpretation. MN and LL: manuscript drafting. ZL, QZ, JZ, and MN: critical revision of the manuscript. All authors contributed to the work approved the final version of the manuscript.

## Footnotes

1
http://www.childhealthdata.org/NSCH

## Supplementary material

Supplementary Figure 1 Multivariable adjusted odds ratios for ADHD according to levels of birth weight on a continuous scale.
