# Supplementary material for: Examining the relationship between birth weight and attention-deficit hyperactivity disorder diagnosis
Source: Front Psychiatry. 2023 May 24;14:1074783. doi: 10.3389/fpsyt.2023.1074783 (PMC10244743; doi:10.3389/fpsyt.2023.1074783)
Supplement: Supplementary file 1 [file Table_1.docx]

**Table S1. Odds Ratios for the Associations Between Birth Weight and ADHD**

|  | Unadjusted model | | Model 1 ^a^ | | Model 2 ^b^ | |
| --- | --- | --- | --- | --- | --- | --- |
|  | OR | 95%CI | OR | 95%CI | OR | 95%CI |
| Birthweight |  |  |  |  |  |  |
| >2500g | Reference |  | Reference |  | Reference |  |
| 1500-2500 | **1.37** | 1.13-1.65 | **1.43** | 1.17-1.74 | **1.32** | 1.04-1.67 |
| <1500 | **1.77** | 1.28-2.45 | **1.83** | 1.32-2.55 | **1.58** | 1.11-2.27 |
| Preterm birth |  |  |  |  |  |  |
| No |  |  |  |  | Reference |  |
| Yes |  |  |  |  | 1.16 | 0.97-1.39 |
| Age of child (years) |  |  |  |  |  |  |
| 3-5 |  |  |  |  | Reference |  |
| 6-12 |  |  | 4.42 | 3.24-6.03 | **4.44** | 3.25-6.06 |
| 13-17 |  |  | 5.50 | 4.04-7.51 | **5.54** | 4.05-7.58 |
| Sex of child |  |  |  |  |  |  |
| Male |  |  |  |  | Reference |  |
| Female |  |  | 0.39 | 0.35-0.44 | **0.39** | 0.35-0.44 |
| Race of the child |  |  |  |  |  |  |
| White |  |  |  |  | Reference |  |
| Hispanic |  |  | 0.61 | 0.50-0.74 | **0.62** | 0.51-0.75 |
| Black/African American |  |  | 0.91 | 0.76-1.08 | 0.93 | 0.78-1.10 |
| Asia |  |  | 0.24 | 0.15-0.38 | **0.24** | 0.15-0.38 |
| Other |  |  | 0.86 | 0.71-1.04 | 0.86 | 0.71-1.05 |
| Household poverty level (%FPL) |  |  |  |  |  |  |
| <100% |  |  |  |  | Reference |  |
| 100-199% |  |  | 0.83 | 0.68-1.01 | **0.82** | 0.68-1 00 |
| 200-399% |  |  | 0.67 | 0.56-0.80 | **0.67** | 0.56-0.81 |
| ≥400% |  |  | 0.62 | 0.52-0.74 | **0.62** | 0.52-0.74 |
| Insurance |  |  |  |  |  |  |
| Yes |  |  |  |  | Reference |  |
| No |  |  | 0.60 | 0.46-0.78 | **0.60** | 0.46-0.79 |
| Household smoking exposure |  |  |  |  |  |  |
| No |  |  |  |  | Reference |  |
| Yes |  |  | 1.54 | 1.33-1.78 | **1.52** | 1.31-1.76 |

1. Model 1 was adjusted for age, sex, race, family poverty, health insurance, smoking, parental mental health.
2. Model 2 was adjusted for preterm birth and the variables in Model 1

The bold value indicated that the P value of the odds ratio was < 0.05.

NBW: normal birth weight; LBW: low birth weight; VLBW: very low birth weight.

**Table S2. Estimates for the Associations Between Birth Weight and ADHD.**

|  | Unadjusted model | | Model 1 ^a^ | | Model 2 ^b^ | |
| --- | --- | --- | --- | --- | --- | --- |
|  | OR 95%CI | P | OR 95%CI | P | OR 95%CI | P |
| Birth weight | 0.99 (0.99-1.00) | 0.025 | 0.99 (0.99-1.00) | <.001 | 0.99 (0.99-1.00) | 0.005 |

One ounce equals 28.35 gram.

a. Model 1 was adjusted for age, sex, race, family poverty, health insurance, smoking, parental mental health.

b. Model 2 was adjusted for preterm birth and the variables in Model 1

NBW: normal birth weight; LBW: low birth weight; VLBW: very low birth weight.

**Table S3. Estimated Odds Ratio of ADHD at Select Birth weight in 60,358 Children in NSCH, 2019 to 2020.**

|  | Unadjusted | | Model 1 | | Model 2 | |
| --- | --- | --- | --- | --- | --- | --- |
| Birth weight (g) | OR | 95%CI |  |  |  |  |
| 2250 | 1.41 | 1.27-1.58 | 1.56 | 1.39-1.74 | 1.32 | 1.16-1.49 |
| 2500 | 1.30 | 1.20-1.41 | 1.41 | 1.29-1.53 | 1.24 | 1.13-1.36 |
| 2750 | 1.18 | 1.12-1.24 | 1.26 | 1.20-1.33 | 1.16 | 1.09-1.23 |
| 3000 | 1.08 | 1.05-1.11 | 1.14 | 1.11-1.17 | 1.09 | 1.05-1.23 |
| 3250 | 1.01 | 1.00-1.03 | 1.05 | 1.03-1.07 | 1.03 | 1.02-1.05 |
| 3500 | Reference | - |  |  |  |  |
| 3750 | 1.02 | 1.00-1.05 | 0.98 | 0.96-1.01 | 0.99 | 0.96-1.01 |
| 4000 | 1.06 | 1.00-1.13 | 0.98 | 0.92-1.04 | 0.98 | 0.92-1.04 |
| 4250 | 1.11 | 1.01-1.22 | 0.98 | 00.89-1.08 | 0.97 | 0.88-1.07 |

a. Model 1 was adjusted for age, sex, race, family poverty, health insurance, smoking, parental mental health.

b. Model 2 was adjusted for preterm birth and the variables in Model 1.
