# Supplementary figures and images for: Examining the relationship between birth weight and attention-deficit hyperactivity disorder diagnosis
Source: Front Psychiatry. 2023 May 24;14:1074783. doi: 10.3389/fpsyt.2023.1074783 (PMC10244743; doi:10.3389/fpsyt.2023.1074783)

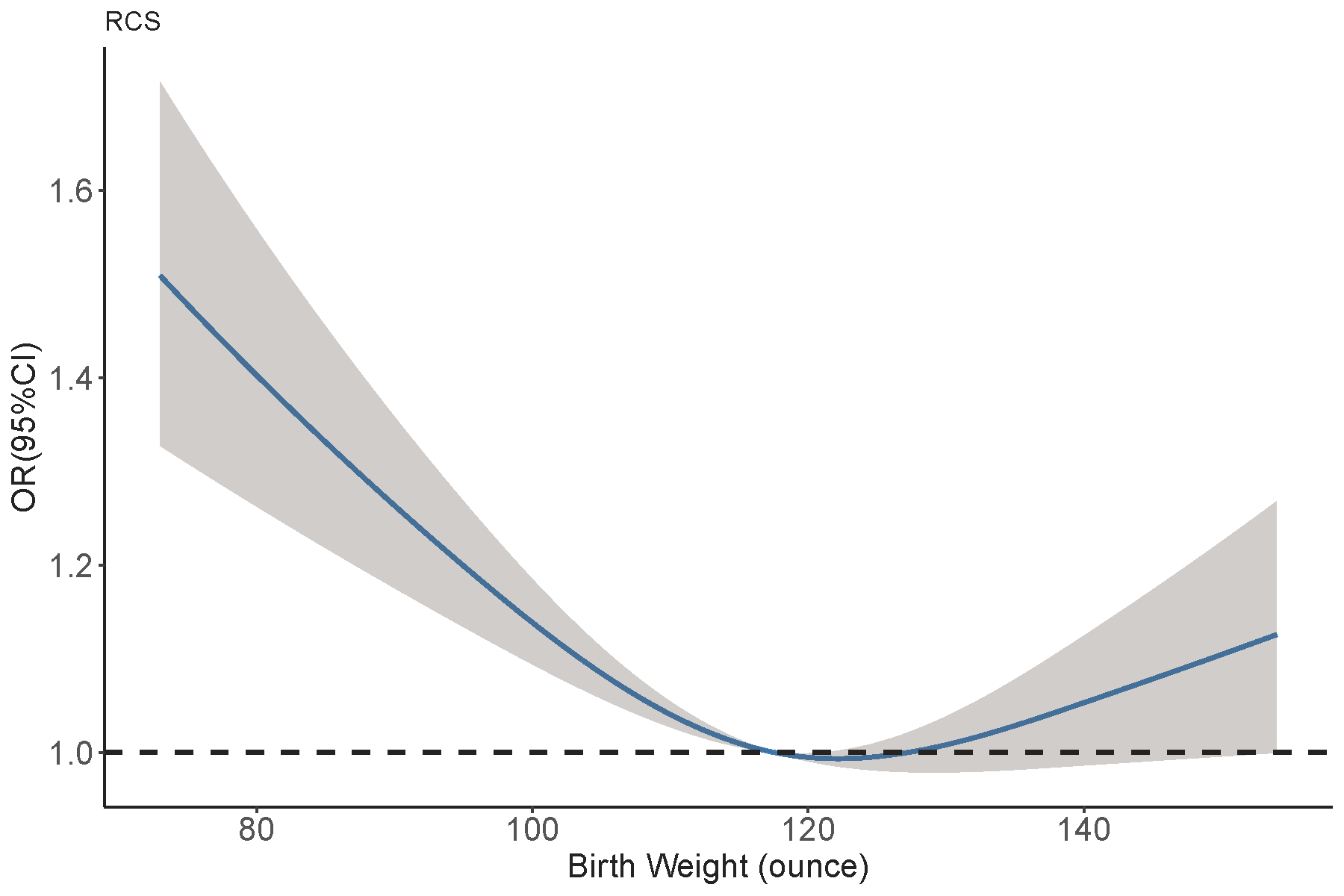

Supplement: Supplementary Figure 1 — Multivariable adjusted odds ratios for ADHD according to levels of birth weight on a continuous scale. [file Image_1.tiff]
